# Conservative Management of Spondylodiscitis after Laparoscopic Sacral Colpopexy: A Case Report and Review of Literature

**DOI:** 10.1055/s-0041-1735153

**Published:** 2021-08-30

**Authors:** Madalena Andrade Tavares, Ana Rita Silva, Marta Gomes de Melo, Márcia Pacheco, Nuno Coutinho, Alexandre Ambrósio, Paula Tapadinhas

**Affiliations:** 1 Gynecology and Obstetrics Service, Hospital Vila Franca de Xira, Lisboa, Portugal; 2 Internal Medicine Service, Hospital Vila Franca de Xira, Lisboa, Portugal; 3 Orthopedics Service, Hospital Vila Franca de Xira, Lisboa, Portugal

**Keywords:** spondylodiscitis, sacral colpopexy, pelvic organ prolapse, urogynecological mesh, discitis, espondilodiscite, colpopexia sacral, prolapso de órgãos pélvicos, tela uroginecológica, discite

## Abstract

Sacral colpopexy is one of the standard procedures to treat apical pelvic organ prolapse. In most cases, a synthetic mesh is used to facilitate the colposuspension. Spondylodiscitis is a rare but potentially serious complication that must be promptly diagnosed and treated, despite the lack of consensus in the management of this complication. We report one case of spondylodiscitis after a laparoscopic supracervical hysterectomy and sacral colpopexy treated conservatively. We also present a literature review regarding this rare complication. A conservative approach without mesh removal may be possible in selected patients (stable, with no vaginal lesions, mesh exposure or severe neurologic compromise). Hemocultures and culture of image-guided biopsies should be performed to direct antibiotic therapy. Conservative versus surgical treatment should be regularly weighted depending on clinical and analytical progression. A multidisciplinary team is of paramount importance in the follow-up of these patients.

## Resumo

A colpopexia sacral é um dos procedimentos padrão para tratar o prolapso de órgãos pélvicos apical. Na maioria dos casos, uma tela sintética é usada para facilitar a colposuspensão. A espondilodiscite é uma complicação rara, mas potencialmente grave, que deve ser prontamente diagnosticada e tratada, apesar da falta de consenso no manejo dessa complicação. Relatamos um caso de espondilodiscite após histerectomia supracervical laparoscópica e colpopexia sacral tratada conservadoramente. Também apresentamos uma revisão da literatura sobre essa complicação rara. Uma abordagem conservadora sem remoção da tela pode ser possível em pacientes selecionadas (estáveis, sem lesões vaginais, exposição da tela ou comprometimento neurológico grave). Hemoculturas e cultura de biópsias guiadas por imagem devem ser realizadas para direcionar a antibioticoterapia. O tratamento conservador versus o cirúrgico deve ser avaliado regularmente, dependendo da progressão clínica e analítica. Uma equipe multidisciplinar é de suma importância no acompanhamento desses pacientes.

## Introduction

Pelvic organ prolapse (POP) is the descent of female pelvic organs (vagina, uterus, bladder, and/or bowel) into or through the vagina. It occurs in between 30 and 76% of women referred to a routine gynecological appointment 1 and between 7 and 19% of them undergo surgical repair. 2

Sacral colpopexy is the gold standard procedure for apical prolapse correction, also allowing simultaneous correction of other compartment defects. The reported long-term success is between 78 and 100%, with recurrence rates of 0 to 18%. 2 The procedure can be performed by laparotomy, laparoscopy, or robotic-assisted laparoscopy.

Sacral colpopexy, either abdominal or minimally-invasive, is usually a safe procedure, but may be associated with complications such as mesh erosion (reported in 3.4% of the cases in abdominal sacral colpopexy and in 2% in robotic-assisted sacral colpopexy), urinary tract infection (10.9%), hemorrhage and/or blood transfusion (4.4%), ileus or small bowel obstruction (2.7%), thromboembolic events (3.3%), urinary injury (4.1%), fistula development, infectious complications (peritonitis, periprosthetic abscess), and spondylodiscitis. 3 4 5 Since some of these complications are uncommon, it is difficult to determine the exact prevalence of these events.

Spondylodiscitis is a rare but serious complication of this procedure characterized by the development of an inflammatory process, infectious or not, that involves vertebral bodies and intervertebral discs of the vertebral spine. 6 It is associated with potentially severe consequences, such as multiple surgeries, prolonged immobilization and hospitalization, neural axis infection, chronic pain, and permanent disability. Considering that the disc space has no direct blood supply, spondylodiscitis develops after hematogenous spread or direct inoculation of microorganisms during invasive spinal surgical procedure. Risk factors for the development of spondylodiscitis, after sacral colpopexy, include infection of other organs (urinary, vaginal, oral, respiratory, and gastrointestinal) and vaginal mesh exposure. 7 Its clinical presentation varies from indolent with no fever to high fever, back pain, mobility limitation, pain radiation to the lower extremities, and vaginal discharge. 8

Most of the reported cases of spondylodiscitis after sacral colpopexy required at least one surgical procedure during the treatment of this complication. 8

We present a case of spondylodiscitis after a laparoscopic supracervical hysterectomy and sacral colpopexy that was treated conservatively with antimicrobial agents. This approach avoided a second surgical procedure with removal of the mesh and eventual recurrence of the prolapse. In addition, we reviewed the literature concerning this type of complication after a sacral colpopexy. We revised the most common surgical route used, types of sutures and meshes, how long after the primary surgery the spondylodiscitis manifested, and if there were any previous infectious episodes associated. Our research also included the type of microorganisms detected in culture samples that could alter the antibiotic therapy normally used in other types of spondylodiscitis.

## Case Report

A 65-year-old multiparous woman with a previous history of type II diabetes mellitus, hypertension, dyslipidemia, bilateral gonarthrosis and gouty arthropathy was referred to our hospital due to complaints of POP. A physical examination revealed a stage 3 prolapse with uterine leading edge according to the POP Quantification system (POP-Q), 9 and she underwent a laparoscopic supracervical hysterectomy with bilateral salpingo-oophorectomy and sacral colpopexy. A nonabsorbable prolene soft mesh (Gynecare Gynemesh, ETHICON, LLC, San Lorenzo, Porto Rico) was attached to the anterior and posterior vaginal walls (one suture anteriorly and two sutures posteriorly) and to the sacral promontory (one suture) using a sterile nonabsorbable braided polyester surgical suture (Ethibond Excel, ETHICON, Inc., Somerville, NJ, USA). At the level of the levator ani muscle, a sterile absorbable polydioxanone monofilament suture (PDS II 2/0, ETHICON, Inc., Somerville, NJ, USA) was used. The mesh was peritonealized using an absorbable synthetic polyglactin braided suture (VICRYL 1/0, ETHICON, Inc., Somerville, NJ, USA). Intravenous cefazolin (2 g) was administered as perioperative antimicrobial prophylaxis. The surgery and the immediate postoperative period were uneventful.

One month after the surgery, the patient presented to the emergency department with complaints of lumbosacral minor pain. She was hemodynamically stable and apyretic. Her urine analysis was positive for leucocytes and proteins, and oral antibiotic ambulatory treatment (amoxicillin/clavulanic acid, according to national guidelines for uncomplicated urinary tract infection) 10 was prescribed. Three months postoperatively, the patient was readmitted with progressive and intense lumbosacral pain radiating to both lower limbs and severe gait limitation. She denied fever, vaginal bleeding, vaginal discharge, urinary complaints, or abdominal pain. The patient was afebrile and her physical examination, including the gynecological exam, showed no abnormalities. There was no evidence of vaginal lesions or mesh exposure. Her blood tests revealed an elevated C-reactive protein (CRP 9.51mg/dL) with a normal white cell count. The remaining blood tests were unremarkable. Her blood, vaginal (including for bacterial vaginosis), and urine cultures did not reveal any atypical or overwhelming microbial growth. A computed tomography (CT) exam showed an anterior interruption of the bone cortical in L5 and S1 with inflammatory reaction involving the intervertebral disc.

**Fig. 1:**
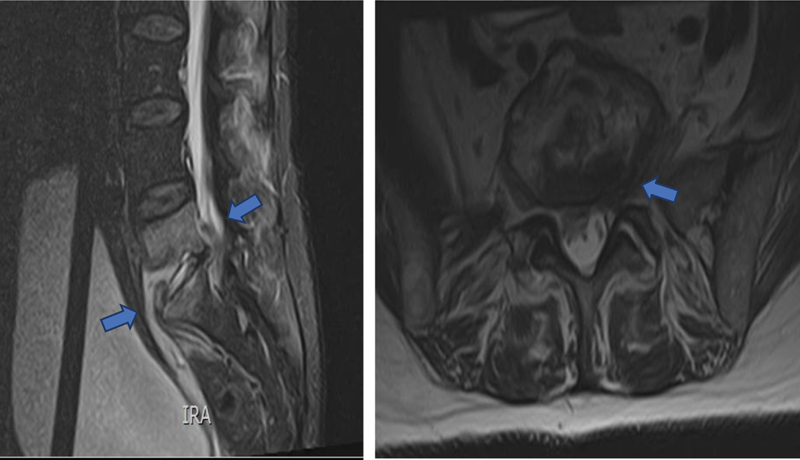
Magnetic resonance imaging before treatment – Accentuated edema of the somatic L5 and S1 sponge with erosive anomalies in the vertebral plates, suggestive of spondylodiscitis. Associated phlegmon with anterior extension to the sacrum and to the posterior peridural region.

A magnetic resonance imaging (MRI) exam revealed an accentuated edema of the somatic L5 and S1 sponge with erosive anomalies in the vertebral plates of these vertebrae, suggestive of spondylodiscitis with associated phlegmon with anterior extension to the sacrum and to the posterior peridural region ( Fig. 1 ).

After multidisciplinary discussion (internal medicine, gynecology and orthopedics), the most likely diagnosis of spondylodiscitis after sacral colpopexy was assumed and a conservative management was implemented (broad-spectrum intravenous antibiotic therapy and physiotherapy).

The patient fulfilled 6 days of intravenous empiric therapy with vancomycin and ceftriaxone (according to international guidelines regarding vertebral osteomyelitis) 11 and then, after discussion with the infectious diseases consultant, continued with 19 days of intravenous amoxicillin and clavulanic acid (to cover anaerobic agents, including *Bacterioides* spp.), with progressive clinical (pain, gait) and analytical (CRP 1.11mg/dL at discharge) improvement. The MRI findings on the 18 ^th^ day of hospitalization remained stable and without neurologic involvement.

The patient was discharged on the 25 ^th^ day of hospitalization, without requiring analgesic medication and medicated with amoxicillin and clavulanic acid per os (until completing a total of 6 weeks of antibiotic therapy with amoxicillin and clavulanic acid) ( Tables 1 and 2 ).

**Table 1:** Characteristics before complication

Characteristic	Data
Mean age in years (range)	60 (41–81)
Surgical route, n (%)	
Laparotomic	19 (36.5)
Laparoscopic	23 (44.2)
Robotic-assisted laparoscopy	8 (15.4)
Not reported	2 (3.8)
Concomitant Procedures, n (%)	
Total hysterectomy	13 (25)
Supracervical hysterectomy	12 (23)
Salpingo-oophorectomy	(17.3)
Rectopexy	7 (13.5)
Burch procedure	3 (5.8)
Sacral anchorage, n (%)	
Synthetic mesh	28 (53.9)
Strips	4 (7.7)
Partially absorbable	4 (7.7)
Biological mesh	1 (1.9)
Direct sutures	2 (3.8)
Unknown	13 (25)
Attachment to the promontory, n (%)	
Nonabsorbable sutures	19 (36.5)
Staples, clips, tacks, or screws	12 (23.1)
Unknown	21 (40.4)

**Table 2:** Characteristics of the complication

Characteristic	Data
Mean time to presentation (range)	12 months (2 days – 8 years)
Initial infection or complication, n (%)	
Urinary infection	9 (17.3)
Dental infection	1 (1.9)
Isolated vaginal discharge	2 (3.8)
Isolated vaginitis	3 (5.8)
Vaginal mesh exposure	2 (3.8)
Vaginal symptoms with vaginal mesh exposure or vaginal lesion	7 (13.6)
Small bowel obstruction	1 (1.9)
Sigmoid diverticulitis	1 (1.9)
Fistula development	1 (1.9)
Not reported	25 (48.1)
Microbiological analysis, n (%)	
Not reported	8 (15.4)
Performed and negative	7 (13.6)
Performed and positive	37 (71)
Management, n (%)	
Antibiotic treatment alone	13 (25)
Antibiotic and surgical treatment	37 (71)
Unknown	2 (3.8)

**Fig. 2:**
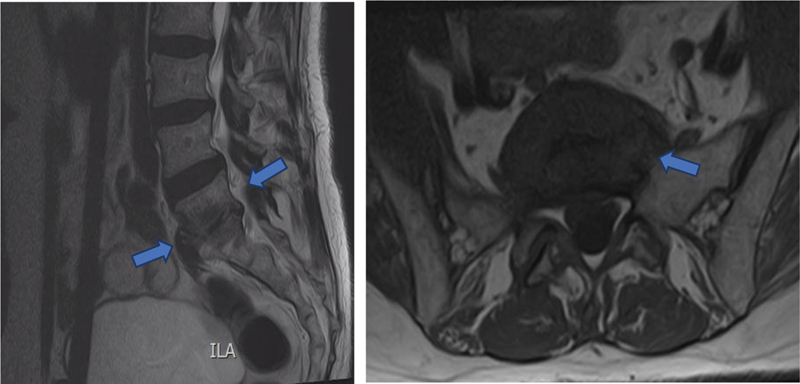
Magnetic resonance imaging after treatment – Resolution of the inflammatory peridural component with post-infection ankylosis signs of L5-S1.

Twenty-four months postsurgery, the patient demonstrated a favorable improvement with minor lumbosacral pain radiating to both proximal lower limbs without needing analgesic medication and with unlimited mobility. An MRI exam revealed resolution of the inflammatory peridural component with postinfection ankylosis signs of L5-S1 ( Fig. 2 ). There was no recurrence of the POP. The patient provided informed consent for the publication of the present case and accompanying images.

## Discussion

### Case Discussion

According to the literature, pyogenic spondylodiscitis can be treated with antibiotics in 50 to 75% of the cases. 12 However, there is little evidence about the correct management of pyogenic spondylodiscitis after abdominal surgery. *Staphylococcus* spp. and *Streptococcus* spp. are the main responsible microorganisms in 50% of all pyogenic spondylodiscitis cases, followed by gram-negative bacilli; therefore, antibiotic therapy with vancomycin and a third- or fourth-generation cephalosporin are usually administered empirically until the results of microbiology are available. 11 The ideal total duration of antibiotic therapy is not well-defined, with some guidelines recommending 6 weeks of total antibiotic therapy (intravenous and oral) with careful review to determine if further treatment is required. 11 13 Other centers recommend intravenous antibiotics for 4 to 6 weeks or until normalization of CPR levels, followed by oral antibiotics until a total of 3 months of treatment. 12

The risks and benefits of surgery should be weighted and adapted to the general status, comorbidities, and response to antibiotic treatment of the patient. Surgery is definitely indicated in case of neural compression, neurologic deficit, progressive deformation or instability or in case of medical treatment failure. 7 14 Surgical treatment often includes mesh removal and/or other orthopedic procedures (laminectomy, discectomy etc).

Although some authors recommend an additional surgery for mesh removal as soon as the diagnosis of spondylodiscitis after sacral colpopexy is confirmed, 15 we opted for a different approach. As our patient did not present severe systemic or neurological symptoms, mesh exposure or vaginal lesions and considering that it was a clean surgery (supracervical hysterectomy without entering the vagina), we decided to initiate a conservative treatment with daily close surveillance. The patient was treated as an inpatient during the 25 days of intravenous antibiotic therapy, which allowed progressive recovery (clinical and analytical) and reinforced the decision of maintaining the conservative treatment. The patient was discharged when her CRP was almost negative and when she became independent from intravenous analgesia.

Even though microbiological cultures (vaginal, urinary, and hematologic) were negative, we verified that, with empirical antibiotic treatment, there was a favorable clinical, analytical and imaging evolution, dismissing the need for a second surgical intervention with eventual mesh removal. This required a close follow-up but without recurrence of pelvic organ prolapse, which was not evaluated in most of the other reports.

However, it would have been recommended to perform an image-guided aspiration or biopsy to establish the exact microorganism causing this complication. Image-guided aspiration or biopsy or intraoperative samples of the infected region remain a very important diagnostic tool that allows pathogen-directed therapy. 11

It is also important to notice that event though permanent braided sutures may be associated with increased infectious risk, these sutures provide greater knot security than monofilament sutures, which, in this type of surgery, reduce the risk of mesh detachment and recurrent prolapsus.

### Literature Review

We performed a PubMed search of the literature on October 4, 2020, using the following terms: *spondylodiscitis* , *discitis* , *osteomyelitis* , *pyogenic* *spondylodiscitis* , *pyogenic* *discitis* , *pyogenic* *osteomyelitis* or *vertebral* *osteomyelitis* and *sacral* *colpopexy* , *pelvic organ prolapse* or *promontofixation* . Additionally, the references of all articles included were confirmed for further information and eventual description of other cases.

Data extraction from PubMed provided a total of 36 abstracts, and further reference search yielded additional 8 articles, gathering a total of 52 cases, 6 7 8 14 15 16 17 18 19 20 21 22 23 24 25 26 27 28 29 30 31 32 33 34 35 36 37 38 39 40 41 42 43 44 45 46 47 48 49 50 51 52 including ours. The summary of the results is presented in Tables 1 and 2 . The list of all studied cases with their detailed characteristics is presented in the Supplementary Table S1 (available online).

In the reported cases of spondylodiscitis after sacral colpopexy, the surgical route was laparotomic in 36.5%, laparoscopic in 44.2%, and robotic-assisted laparoscopy in 15.4%. Sacral anchorage was performed with synthetic mesh (nonabsorbable or partially absorbable) or strip in the majority of the cases (63.5%). Biologic meshes and direct sutures were used in 1.9 and 3.8% of the surgeries, respectively. In 25% of all cases, the type of sacral anchorage was not specified. The attachment to the promontory was made with sutures in 36.5% (all nonabsorbable sutures) and with staples, clips, tacks or screws in 23% of the cases. In 40.4% of the cases, information about attachment was lacking.

There is still debate regarding the best mesh and suture material to use, but, as observed, nonabsorbable material is the most frequently used, with better results in terms of prolapse resolution. 53

The time between surgery and clinical presentation of spondylodiscitis ranged from a few days after surgery up to 8 years, but the majority presented with symptoms < 5 months after surgery (75%).

Spondylodiscitis was preceded by a documented infection or other complication in 52% of the cases. Previous urinary or dental infection was reported in 19.2% of the cases. Isolated vaginal discharge or vaginitis were reported in 9.6%, while mesh erosion without vaginal symptoms occurred in 3.8% of the cases. In 13.5% of the reported cases, there was a combination of vaginal symptoms (vaginitis/vaginal discharge) with vaginal mesh exposure or vaginal lesion (vaginal ulcer/vaginal apex opening) – in one of these cases, there was a concomitant urinary tract infection and, in another case, small bowel obstruction was reported. Urinary tract infection as a previous infection occurred more frequently in women who underwent supracervical hysterectomy (5 cases in 23 hysterectomies) than in those who underwent total hysterectomy (0 cases in 25 total hysterectomies). On the other hand, vaginal symptoms and mesh exposure seem to occur more frequently in total hysterectomies (6 in 25 hysterectomies versus 1 in 23 supracervical hysterectomies).

Other events that preceded the presentation of spondylodiscitis were: small bowel obstruction (two cases – one of them in association with vaginal discharge and mesh erosion), sigmoid diverticulitis (one case), and a fistula development from rectopexy to the anterior vertebral ligament (one case).

In 44 of the 52 studied cases, there were reports on the microbiological analysis of collected samples (blood, urine, vaginal, image-guided biopsies/aspirations of infection site, surgical samples of the mesh or the surrounding tissue). Of these, 13.5% ( *n* = 7) had negative cultures. From the 44 cases that reported microbiological analysis, a single microorganism was found in 47.7% ( *n* = 21), and polymicrobial infections were present in 36.4% ( *n* = 16).

The most prevalent microorganism detected were: *Bacteroides* spp. (19.2%), *Staphylococcus aureus* (15.4%), *Escherichia coli* (15.4%), and *Enterococcus* spp. (13.5%). The prevalence of infections related to *Streptococcus* spp. and *Staphylococcus* spp. was 32.7%.

In 15 cases, there was information about peripherical cultures (blood, urine, and vaginal swab), documenting 2 negative and 13 positive cases. In this type of microbiological culture (31% polymicrobial infections), the most prevalent microorganisms detected were: *Bacteroides* spp. (38.5%) followed, in equal prevalence of 31% by *S. aureus, E. coli* and *Streptococcus* spp.

Regarding cultures from the infection site or local cultures (image-guided biopsies/aspirations or intraoperative samples including mesh culture), the review revealed 6 negative and 33 positive cultures. In the positive cultures group, *S. aureus* was the most prevalent microorganism (21%), followed by *Bacteroides* spp. (18%), *Enterococcus* spp. (15%) and *Pseudomonas aeruginosa* (15%). The prevalence of infections related to *Streptococcus* spp. and/or *Staphylococcus* spp. was 46% in peripherical cultures and 39% in cultures from the infection site.

Unfortunately, cultures from the infection site are not always consistent with peripherical cultures. From all the positive peripherical cultures, only in 62% (8 cases) the microorganism detected was the same as in the culture from the infection site (7 cases of positive hemocultures and 1 case of positive uroculture). Even for positive hemocultures, 36% did not correspond to the same microorganism of the local culture. Therefore, although peripherical cultures (mainly hemocultures) are strongly recommended to detect all possible microorganisms responsible for this infection, they will not be enough to narrow the empirical antibiotic therapy. The most recommended diagnostic tool for microorganism detection is image-guided biopsy/aspiration or culture from intraoperative samples.

During our review, we noticed that the prevalence of infections due to *Streptococcus* spp. and *Staphylococcus* spp. as a group was lower than the one reported in the literature for pyogenic spondylodiscitis (50%), and other microorganisms such as *Bacteroides* spp., *E. coli* and *Enterococcus* spp. had a relevant prevalence. This emphasizes the paramount importance of sample collection for microbiological analysis and antibiotic therapy decision and of the need to include anaerobe coverage if an empirical antibiotic therapy is started.

In addition to our case, the research revealed 12 cases that were treated only with antibiotics. In 11 of these cases, there was a positive hemoculture or site infection culture (in 1 case there is no information about cultures), with *Bacteroides* spp., *S. aureus* and *P. aeruginosa* being the most common pathogens identified. The type of antibiotic treatment in this group was heterogenous, and its duration varied from 4 weeks up to 6 months (4 weeks in 9%; 8 to 12 weeks in 64%; and > 12 weeks in 27% of the patients).

Most cases of surgical treatment (performed in 37 cases) involved mesh or bone anchors removal (81% of all cases treated surgically; 57.7% of all reported cases). Other common associated procedures were discectomy, laminectomy, and abscess debridement. In some cases, the patient underwent > 1 surgery to treat the complication. The duration of the antibacterial treatment in surgically treated patients was not always revealed (only in 43.6% of the cases). In the described cases, it was maintained for 2 to 14 weeks (< 6 weeks in 23.5%; 6 to 8 weeks in 35.3%; 8 to 12 weeks in 17.7%; and > 12 weeks in 23.5% of the patients), while antifungal therapy was prolonged for 12 months in 1 patient.

Recommendations to prevent spondylodiscitis associated with sacral colpopexy have been suggested. During this procedure, the step of fixation of the mesh to the sacral promontory (defined as the most superior point on the anterior surface of S1) is of paramount importance not only for diminishing the risk of hemorrhage but also to avoid making a very deep stitch or risking incorrect location. 54

The suture should be attached to the anterior longitudinal ligament keeping in mind that its maximum thickness is 2 mm. Additionally, it is of utmost relevance to avoid placing the suture in the region of the disc space. To achieve this, the surgeon should directly visualize the ligament as well as palpate the hard-bony part instead of the spongy disc (easier at laparotomy than in minimally invasive procedures). Good et al. 54 reported the existence, in the supine position, of an acute angle of descent (average 60°) between the anterior surfaces of L5 and S1. Consequently, the most prominent structure in the presacral space is the L5-S1 disc and not the “true” sacral promontory. Thereby, if the surgeon does not acknowledge this fact, it is not infrequent for the mesh to be attached to the intervertebral disc. 54

## Conclusion

We analyzed 52 cases of spondylodiscitis after sacral colpopexy. Being a rare complication of sacral colpopexy, there should be a high index of suspicion for spondylodiscitis after this type of procedure. In 52% of the cases, there was a previous documented infection (mainly vaginal or urinary) or other complication (mesh exposure), and these patients should be followed-up closely to precociously detect this possible complication. Back pain associated with elevated CRP after this procedure should lead us to further investigation to achieve a prompt diagnosis and initiate adequate treatment. A conservative approach without mesh removal may be possible with vigilant attention. However, hemocultures and image-guided biopsy/aspiration of the infection site should not be neglected, since the microorganisms causing this complication may be different from the typical *Staphylococcus* spp. and *Streptococcus* spp. responsible for 50% of other types of spondylodiscitis. If empirical treatment is prescribed, it should also cover anaerobe, to cover for *Bacteroides* spp., which is one of the most common microorganisms detected in this complication. The patient should be admitted for attentive surveillance and intravenous antibiotic therapy and analgesia. The authors recommend conservative treatment in stable patients without vaginal mesh exposure, vaginal lesion, neural compression, neurologic deficit, progressive deformation, or instability. If there is a favorable recovery of clinical symptoms and analytical parameters, conservative treatment should be maintained with prolonged antibiotic therapy for at least 6 weeks. On the other hand, neurological aggravation or conservative treatment failure should redirect the approach for a surgical treatment. It is also of paramount importance that a multidisciplinary team is implicated in the follow-up of these patients.
