# Supplementary material for: Conservative Management of Spondylodiscitis after Laparoscopic Sacral Colpopexy: A Case Report and Review of Literature
Source: Rev Bras Ginecol Obstet. 2021 Aug 30;43(7):570–7. doi: 10.1055/s-0041-1735153 (PMC10303985; doi:10.1055/s-0041-1735153)
Supplement: Supplementary file 1 — Supplementary Material [file 10-1055-s-0041-1735153-s200419.pdf]

Supplementary Material 1 Reported cases of spondylodiscitis after sacral colpopexy

	Author	Age	Primary Surgical Procedure	Mesh Type	Promontory Suture Type	Time to Presentation	Initial infection or Complication	Cultures	Antibiotic regimen	Surgical Treatment
1	Tavares et al.	65	Laparoscopic supracervical hysterectomy, bilateral salpingo-oophorectomy and sacral colpopexy	Nonabsorbable Prolene soft mesh	Polyester nonabsorbable, braided	1 month	Urinary tract infection	Blood, Vaginal and Urine cultures: Negative	- Vancomycin, and Ceftriaxone (6 days IV)<br>- Amoxicillin/ Clavulanic acid (19 days IV)<br>- Amoxicillin/ Clavulanic acid (23 days *per os*)	Not performed
2	Qu et al.[16]	46	Laparoscopic sacral colpopexy	Polypropylene mesh	NR	4 months	NR	Cultured mesh: *Escherichia coli*	- Imipenem/Cilastatin 2 weeks	Mesh removal<br>Debridement, laminectomy, spinal canal decompression, bone grafting internal fixation via pedicle screw placement
3	Miksić et al.[17]	81	Laparoscopic sacral colpopexy	NR	Titanium bone anchors	18 weeks	NR	Blood cultures: *Bacteroides fragilis*	- Amoxicillin/ Clavulanic (4 days IV)<br>- Metronidazole and Ceftriaxone (3 weeks IV)<br>- Metronidazole and ciprofloxacin (8 days *per os*)<br>- Clindamycin (6 months *per os*)	Not performed
4	Sato et al.[18]	76	Laparoscopic supracervical hysterectomy and sacral colpopexy	Unspecified mesh	NR	2 days	Vaginal discharge	Intraoperative smears: *Bacteroides fragilis*, *Petoniphilus* spp, *Prevotella* spp.	- Minocycline *per os*	Abscess drainage<br>Mesh removal
5	Boyd et al.[19]	71	Robotic sacral colpopexy, bilateral salpingo-oophorectomy, and lysis of adhesions	Polypropylene mesh	Polytetrafluoroethylene microporous, monofilament suture	6 weeks	Vaginal candidiasis	Mesh culture: *Candida albicans*	- Vancomycin, ceftriaxone, and metronidazole, Fluconazole (IV)<br>- Ceftriaxone and vancomycin (6 week IV)<br>- Fluconazole (12 months *per os)*	Adhesiolysis<br>Mesh removal
6	Müller et al.[8]	60	Laparoscopic posterior rectopexy	No mesh	Permanent Monofilament sutures	2 weeks	Fistula from the dorsal rectopexy to the anterior vertebral ligaments and intervertebral dis L5/S1	Epidural samples: *Pseudomonas aeruginosa*, *Enterococcus faecium*, *Enterococcus faecalis*	Unknown	Short colonic segment resection
7	Gungor et al.[15]	52	Laparoscopic Total hysterectomy, bilateral salpingo-oophorectomy and sacral colpopexy.	Polypropylene mesh	Polypropylene sutures	3 months	NR	Cultured mesh: Negative	- Ceftriaxone, Clindamycin (4 weeks IV)<br>- Vancomycin, Ciprofloxacin, Metronidazole	Mesh removal
8	Gupta et al.[20]	NR	Robotic assisted Laparoscopic Sacral colpopexy or Sacral hysteropexy (not specified)	Polypropylene mesh	Polytetrafluoroethylene microporous, monofilament suture<br>Polyester nonabsorbable, braided suture<br>polydioxanone absorbable monofilament suture	NR	NR	NR	Unknown	Surgical treatment not specified
9	Pasquer et al.[21]	76	Laparoscopic Sacral cystopexy and rectopexy	Polypropylene strips	Nonabsorbable polyester suture	1 month	NR	Blood culture: *Escherichia coli*	- Ceftriaxone, Ofloxacin	Hartmann's procedure

(*Continued*)

 © 2021. Federação Brasileira de Ginecologia e Obstetrícia. All rights reserved.

Supplementary Material 1 (*Continued*)

	Author	Age	Primary Surgical Procedure	Mesh Type	Promontory Suture Type	Time to Presentation	Initial infection or Complication	Cultures	Antibiotic regimen	Surgical Treatment
10	Feng et al.[22]	64	Robotic-assisted laparoscopic supracervical hysterectomy with sacral colpopexy. Sub-urethral sling	NR	NR	1 month	NR	Spinal aspiration: *Bacteroides fragilis*	- Ertapenem (IV 12 weeks)	Mesh removal
11	Jenson et al.[14]	67	Laparoscopic-assisted vaginal hysterectomy, bilateral salpingo-oophorectomy and sacral colpopexy.	Polypropylene mesh	NR	4 months	Vaginal Mesh Exposure	CT-guided biopsy: *Enterococcus faecalis, vancomycin-resistant Enterococcus gallinarum, Bacteroides fragilis*	- Vancomycin and Metronidazole<br>- Piperacillin/ Tazobactam	Mesh Removal
12	Núñez–Pereira et al.[23]	80	Laparotomic sacral colpopexy	NR	NR	7 years	NR	Intraoperative smears: *Enterococcus faecium, Enterococcus faecalis*	- Ceftriaxone, Metronidazole<br>- Vancomycin<br>- Linezolid, rifampicin, imipenem<br>- Vancomycin, Ceftriaxone, Rifampicin	Mesh removal<br>Abscess debridement<br>Lumbar fusion<br>L1-L4 decompression<br>Rectosigmoidal resection<br>Protective loop ileostomy
13	Brito et al.[24]	61	Laparoscopic supracervical hysterectomy, bilateral salpingo-oophorectomy and sacral colpopexy. Sub-urethral sling	NR	NR	12 days	NR	Urine culture: Negative<br>Blood Culture: Group-B *Streptococcus*<br>Mesh culture: *Staphylococcus aureus*	- Ceftriaxone and Amikacin<br>- Gentamycin and Amoxicillin<br>- Levofloxacin and Amoxicillin	Mesh removal
14	Tymchak et al.[25]	61	Vaginal Hysterectomy. Laparotomic sacral colpopexy	NR	NR	2 months	NR	CT-guided biopsy: Negative	- Vancomycin (6 weeks IV)<br>- Moxifloxacin (8 weeks *per os*)	Mesh removal
15	Api et al.[6]	65	Laparotomic total hysterectomy and sacral colpopexy	Polypropylene mesh	NR	53 days	NR	Cultured mesh: Negative	- Ceftriaxone, Metronidazole	Mesh removal
16	Api et al.[6]	53	Laparotomic total hysterectomy and sacral colpopexy	Polypropylene mesh	NR	6 days	NR	Cultured mesh: Negative	- Clindamycin, Gentamycin	Mesh removal
17	Vujovic et al.[26]	50	Laparoscopic ventral mesh rectopexy	Biological mesh	Titanium screws	6 weeks	NR	Blood cultures: Negative<br>Intra-operative smear: Negative	Unknown	Surgical screw removal
18	Kdous et al.[27]	53	Laparoscopic sacral colpopexy	Prolene strips	Spiral Staples Tacker type	15 days	NR	Blood cultures: Negative<br>Epidural samples: *Pseudomonas aeruginosa*	- Ciprofloxacin and Piperacillin/ Tazobactam (8 weeks IV)<br>- Rifampicin and Ciprofloxacin (6 weeks *per os*)	Mesh and staples removal
19	Arsene et al.[28]	NR	Total hysterectomy and sacral colpopexy	Polyester mesh	Non absorbable suture	1 month	Vaginal mesh exposure	NR	Unknown	Mesh removal
20	Propst et al.[7]	55	Laparotomic total hysterectomy and sacral colpopexy	Macropore polyester mesh with a reabsorbable collagen barrier	Polypropylene sutures	3 years	Vaginitis<br>Mesh erosion	Intra-operative smear: *Prevotella* spp, *Bacteroides* spp, *Streptococcus viridans*	Unknown antibiotherapy for 6 weeks	Mesh Removal<br>Abscess debridement
21	Propst et al.[7]	66	Robotic assisted laparoscopic Sacral colpopexy and ventral rectopexy	Polypropylene mesh and porcine small intestinal mucosa graft	Monofilament permanent suture	8 weeks	Vaginal discharge	Epidural sample: *Bacteroides fragilis*	- Vancomycin, Ciprofloxacin<br>- Ertapenem and Vancomycin (6 weeks IV)	Mesh removal<br>Laminectomy<br>Discectomy

 © 2021. Federação Brasileira de Ginecologia e Obstetrícia. All rights reserved.

Supplementary Material 1 (*Continued*)

	Author	Age	Primary Surgical Procedure	Mesh Type	Promontory Suture Type	Time to Presentation	Initial infection or Complication	Cultures	Antibiotic regimen	Surgical Treatment
22	Propst et al.[29]	81	Laparoscopic rectopexy	NR	NR	3 weeks	NR	Blood cultures and CT-guided biopsy: *Pseudomonas aeruginosa*	- Piperacillin/ Tazobactam (2 weeks)<br>- Ceftazidime (IV)<br>- Ciprofloxacin (12 weeks *per os*)	Not performed
23	Anand et al.[30]	70	Robotic assisted laparoscopic supracervical hysterectomy and sacral colpopexy. Sub-urethral sling.	Partially Absorbable Lightweight Mesh	Polytetrafluoroethylene microporous, monofilament suture	3 months	Urinary Tract Infection	Urine Culture: *Escherichia coli; Enterococcus faecalis, Leuconostoc lactis, Candida galbrata*<br>Vertebral spine cultures: *Candida galbrata*	- Ampicillin and sulbactam<br>- Vancomycin and Piperacillin/ Tazobactam<br>- Vancomycin and ertapenem (4 weeks IV and 2 weeks *per os*)<br>- Antifungal antibiotics (12 weeks IV)	Mesh removal<br>Anterior discectomy
24	Apostolis et al.[31]	66	Laparoscopic total hysterectomy, bilateral salpingo-oophorectomy and sacral colpopexy. Sub-urethral sling.	Monofilament Polypropylene Mesh	Protack titanium coil devide	2 weeks	Infected upper incision – dental extraction	Blood culture: *Bacteroides fragilis*<br>Mesh culture: *Enterobacter aerogenes*	- Piperacillin/ Tazobactam (IV)<br>- Meropenem and Vancomycin (IV<br>- Ertapenem IV, Micafungin IV, Daptomycin IV, Metronidazole *per os* (5 weeks)<br>- Ertapenem (6 weeks IV)	Mesh removal<br>Laminectomy and debridement of epidural phlegmon
25	Roth et al.[32]	76	Laparoscopic sacral colpopexy and Burch colposuspension.	Nonabsorbable Polyester Fiber Mesh	NR	7.5 years	Vaginal discharge and mesh erosion<br>Small bowel obstruction	Intra-operative smear: *Streptococcus viridans, Streptococcus intermedius*	- Vancomycin and Piperacillin/ Tazobactam (IV)<br>- Ertapenem (2 weeks IV)	Mesh removal<br>Abscess debridement
26	Voelker et al.[33]	58	Laparotomic radical colpectomy and lymphadenectomy. Vaginal reconstruction with a sigmoid interpolate. Sacral colpopexy	Polypropylene Mesh	NR	3 years	NR	Intra-operative smear: *Proteus mirabilis, Morganella morganii, Staphylococcus warneri, Enterococcus faecalis*	- Ciprofloxacin and rifampicin (3 months)	Removal of neovagina, debridement, excision of intervertebral disk with bone graft replacement<br>Dorsal instrumentation of L5-S1 segments
27	Rajamaheswari et al.[34]	42	Laparotomic total hysterectomy and sacral colpopexy	Monofilament macropore polypropylene mesh	NR	4 weeks	Vaginal discharge<br>Mesh Erosion	NR	Unknown	Mesh removal
28	Grimes et al.[35]	63	Robotic-assisted laparoscopic sacral colpopexy, retropubic tension free vaginal tape	Polypropylene Mesh	Non-absorbable multifilament polyester and polytetrafluoroethylene suture	4 months	Yeast vaginitis	Intraoperative smears: *Candida albicans*	Unknown	Mesh removal and debridement of infected area<br>Exposure of posterior spine with screw placement<br>Anterior L4-L5 discectomies and corpectomies<br>Posterior iliac screws and spine fusion
29	Draaisma et al.[36]	55	Laparoscopic ventral sacral rectopexy	NR	NR	3 months	NR	NR	Unknown antibiotherapy for 4 weeks	Not performed
30	Draaisma et al.[36]	45	Laparoscopic ventral sacral rectopexy	NR	Tacks	1 month	NR	NR	Unknown	Mesh removal<br>Deviating colostomy
31	Collins et al.[37]	74		Polypropylene mesh	NR	8 years	Urinary tract Infection		Unknown	

(*Continued*)

 © 2021. Federação Brasileira de Ginecologia e Obstetrícia. All rights reserved.

Supplementary Material 1 (*Continued*)

	Author	Age	Primary Surgical Procedure	Mesh Type	Promontory Suture Type	Time to Presentation	Initial infection or Complication	Cultures	Antibiotic regimen	Surgical Treatment
			Laparotomic sacral colpopexy					Blood culture: *Bacteroides fragilis*<br>Tissue culture: *Vancomycin-resistant Enterococcus*		Mesh removal<br>Abscess debridement
32	Muffly et al.[38]	46	Robotic assisted laparoscopic total hysterectomy and sacral colpopexy.	Nonabsorbable Prolene soft mesh	Braided Polyester Permanent suture	9 months	Vaginal discharge<br>Vaginal Apex opening	Blood and vaginal tissue cultures: Negative<br>Mesh culture: *Staphylococcus aureus*	Vancomycin and Ampicillin-sulbactam (6weeks IV)	Mesh Removal<br>Discectomy
33	Dalwai et al.[39]	NR	Laparoscopic Sacral colpopexy	Unspecified mesh	Titanium tacks	4 weeks	NR	NR	Unknown	Unknown
34	Dalwai et al.[39]	NR	Laparoscopic Sacral colpopexy	Unspecified mesh	Stainless steel screws	4 weeks	NR	NR	Unknown	Unknown
35	Nosseir et al.[40]	55	Robotic assisted laparoscopic supracervical hysterectomy and sacral colpopexy. Sub-urethral sling.	Soft Polypropylene mesh	Titanium Tack	6 weeks	NR	CT needle aspiration : *Staphylococcus aureus*	Unknown antibiotherapy for 8 weeks	Not performed
36	Descargues et al.[41]	54	Laparoscopic supracervical hysterectomy, bilateral salpingo-oophorectomy and sacral colpopexy. Sub-urethral sling.	Polyester strips	Braided polyethylene coated with silicone suture	18 months	Sigmoid diverticulitis	CT-guided biopsy: *Pseudomonas aeruginosa*	Unknown antibiotherapy for 2 weeks Intravenous and 3 months *per os*	Not performed
37	Downing et al.[42]	52	Laparoscopic sacral colpopexy	Nonabsorbable Polyester Fiber Mesh	Polytetrafluoroethylene microporous, monofilament suture	14 months	Yeast vaginitis	Fine needle aspiration of L5-S1 space: *Staphylococcus aureus*	- Nafcillin, Ceftriaxone, Metronidazole (IV)<br>- Vancomycin (6 weeks IV), Levofloxacin and Metronidazole (2 weeks *per os)*	Total abdominal hysterectomy, bilateral salpingo-oophorectomy. Mesh removal
38	Taylor et al.[43]	64	Laparoscopic assisted vaginal hysterectomy, bilateral salpingo-oophorectomy and sacral colpopexy.<br>Burch Colposuspension.<br>Posterior colporrhaphy.	Nonabsorbable Polyester Fiber Mesh	Polyester nonabsorbable braided suture	8 months	Vaginal discharge<br>Vaginal mesh erosion	Blood and Bone cultures: *Methicillin-resistant Staphylococcus aureus*	- Vancomycin and Clindamycin (2 days IV)<br>- Vancomycin (6 weeks IV)	Mesh removal<br>Laminectomy
39	Hart et al.[44]	42	Laparotomic total hysterectomy with bilateral salpingo-oophorectomy and sacral colpopexy.<br>Pubovaginal sling	Polytetrafluoroethylene graft	NR	7 months	Vaginal discharge<br>Vaginal mesh erosion	Blood, Urine and vaginal cultures: Negative	- Ampicillin, Sulbactam, Vancomycin and Metronidazole (4 weeks IV)	Mesh removal Laparotomy with sacral debridement and partial vaginectomy
40	Salman et al.[45]	59	Laparotomic sacral colpopexy	Prolene Mesh	Staples	4 months	NR	NR	Unknown	Abscess debridement, Posterior stabilization
41	Beloosesky et al.[46]	74	Laparotomic sacral colpopexy	Polytetrafluoroethylene graft	Nickle and Titanium clips	7 weeks	Urinary Tract Infection	Blood culture: Negative<br>Urine Culture: *Escherichia coli*<br>Tissue culture: *Staphylococcus epidermidis*	- Gentamycin (6 days IV)<br>- Cloxacillin and Ciprofloxacin<br>- Ciprofloxacin and Vancomycin	Laminectomy
42	Kapoor et al.[47]	63		Polypropylene mesh	Titanium staples	3 weeks	NR			Not performed

 © 2021. Federação Brasileira de Ginecologia e Obstetrícia. All rights reserved.

Supplementary Material 1 (*Continued*)

	Author	Age	Primary Surgical Procedure	Mesh Type	Promontory Suture Type	Time to Presentation	Initial infection or Complication	Cultures	Antibiotic regimen	Surgical Treatment
			Laparoscopic sacral colpopexy					Blood culture: Group B *Streptococcus*	Unknown antibiotherapy for 8 weeks	
43	Cosson et al.[48]	45	Laparoscopic sacral colpopexy and Burch Colposuspension	Prolene strips	Spiral Staples Tacker type	2 years	Urinary Tract Infection	CT-guided biopsy: *Escherichia coli*	- Penicillin M and Quinolone (8 weeks IV)	Mesh Removal
44	Gertzbein et al.[49]	57	NR	NR	NR	"Few days"	Urinary Tract Infection<br>Vaginal discharge<br>Vaginal apex ulcer	Urine culture: *Enterococcus* spp.<br>Tissue culture: Negative	- Penicillin V potassium	Removal of bone anchors Discectomy. Interbody fusion
45	Weidner et al.[50]	56	Laparotomic total hysterectomy and sacral colpopexy	Braided Polyester mesh	Non absorbable braided polyester suture	4 months	NR	Blood culture and CT-guided biopsy: *Streptococcus viridans, Bacteroides fragilis.*<br>Urine culture: *Escherichia coli*	- Clindamycin, Gentamycin and Ampicillin (12 weeks IV)	Not performed
46	Weidner et al.[50]	67	Laparotomic sacral colpopexy<br>Posterior colporrhaphy	Braided Polyester mesh	Non absorbable braided polyester suture	5 years	NR	CT-guided biopsy: *Pseudomonas aeruginosa*	- Piperacillin and Gentamycin (4 weeks IV)<br>- Ciprofloxacin (4 weeks *per os)*	Not performed
47	Cranney et al.[51]	72	Laparotomic sacral colpopexy	Polypropylene mesh	NR	4 weeks	Urinary tract infection	Blood culture: Group B *Streptococcus, Staphylococcus aureus, Streptococcus anginosus, Bacteroides fragilis*<br>Tissue culture: Group B *Streptococcus, Staphylococcus aureus*	Unknown	Mesh removal discectomy, spinal fusion
48	Cailleux et al.[52]	59	Laparotomic supracervical hysterectomy and sacral colpopexy	NR	NR	2.5 months	Pyelonephritis	Blood culture: Negative<br>CT-guided biopsy: *Escherichia coli*	Unknown antibiotherapy 30 days IV and 2 months *per os*	Not performed
49	Cailleux et al.[52]	56	Laparotomic supracervical hysterectomy and sacral colpopexy	NR	NR	3.5 months	NR	Blood culture: *Staphylococcus aureus*<br>CT-guided biopsy: *Staphylococcus epidermidis*	Unknown antibiotherapy 30 days IV and 2 months *per os*	Not performed
50	Cailleux et al.[52]	55	Laparotomic supracervical hysterectomy and sacral colpopexy	NR	NR	2 months	Intra-operative cystostomy<br>Urinary Tract Infection	Blood culture: *Staphylococcus aureus*<br>CT-guided biopsy: *Staphylococcus aureus*	Unknown antibiotherapy 30 days IV and 2 months *per os*	Not performed
51	Cailleux et al.[52]	41	Laparotomic supracervical hysterectomy and sacral colpopexy	No mesh	Polyester nonabsorbable braided suture	3 months	Urinary tract infection	Blood culture: *Bacteroides fragilis, Proteus mirabilis*<br>CT-guided biopsy: *Proteus mirabilis*	Unknown antibiotherapy 30 days IV and 2 months *per os*	Not performed
52	Cailleux et al.[52]	54	Laparotomic supracervical hysterectomy and sacral colpopexy.	Polyester mesh	Polyester nonabsorbable braided suture	1.5 months	Small Bowel Obstruction	Blood and tissue culture: *Escherichia coli*	Unknown	Mesh removal

NR - Not reported; IV - intravenous.

 © 2021. Federação Brasileira de Ginecologia e Obstetrícia. All rights reserved.
